# Draft Genome Sequence of *Streptomyces incarnatus* NRRL8089, which Produces the Nucleoside Antibiotic Sinefungin

**DOI:** 10.1128/genomeA.00715-15

**Published:** 2015-07-09

**Authors:** Kenshiro Oshima, Masahira Hattori, Hitomi Shimizu, Koji Fukuda, Michiko Nemoto, Kenji Inagaki, Takashi Tamura

**Affiliations:** a Center for Omics and Bioinformatics, Graduate School of Frontier Sciences, The University of Tokyo, Tokyo, Japan; b Graduate School of Life and Environmental Sciences, Okayama University, Okayama, Japan; c PRESTO, Japan Science and Technology Agency, Kawaguchi, Japan

## Abstract

A draft genome sequence of *Streptomyces incarnatus* NRRL8089, which produces the nucleoside antibiotic sinefungin, is described here. The genome contains 8,897,465 bp in 76 contigs and 8,266 predicted genes. Interestingly, the genome encodes an open reading frame for selenocysteine-containing formate dehydrogenase-O and the selenoprotein biosynthetic gene cluster *selABCD*.

## GENOME ANNOUNCEMENT

*Streptomyces* is a soil bacterium known for its production of secondary metabolites, such as antibiotics. Certain *Streptomyces* species produce nucleoside antibiotics with potent activity against fungi, viruses, and protozoa. The nucleoside antibiotic sinefungin was isolated from the culture broth of *Streptomyces incarnatus* 8089 (1). This nucleoside antibiotic exhibits antifungal (2) and antiviral activity (3), as well as potent activity against a number of protozoal parasites, including malaria and trypanosome (4–7). This secondary metabolite has also been a target for enhancing the production through protoplast regeneration (8) and *rpoB* mutation (9).

*De novo* shotgun sequencing was performed using a Roche Genome Sequencer FLX. A shotgun library and 8-kb mate pair library were obtained according to the manufacturer’s protocols. Total reads of 715,821 fragments encompassing 460,270,476 bp were assembled using the Newbler version 2.8. The resulting DNA scaffolds were further analyzed using Rapid Annotations with Subsystems Technology (RAST) (10); the NCBI Prokaryotic Genome Annotation Pipeline (11) was also used for gene annotation for submission to GenBank. tRNAscan-SE revealed 68 tRNAs representing all 20 standard amino acids as well as selenocysteine. The draft genome sequence of *Streptomyces incarnatus* NRRL8089 was estimated to be 8,878,066 bp, representing 50× coverage. The genome has a G+C content of 71.71%. The assembled genome consists of 76 contigs, including the longest contig of 763,868 bp.

antiSMASH (12) predicted 32 gene clusters, including genes for type I, II, and III polyketide synthetases, nonribosomal peptide synthetases, and other biosynthetic genes for siderophores, terpenes, butyrolactones, lantibiotics, melanine, and l-ecognine. An interesting metabolic characteristic was noticed in the genome that includes genes for anaerobic energy metabolism involving l-selenocysteine-containing formate dehydrogenase (FDH-0). The in-frame opal codon UGA was directly followed by a selenocysteine insertion sequence (SECIS) element, and the prokaryotic selenosome genes *selA, selB, selC,* and *selD* were also present in the genome as a cluster in the vicinity of the selenoprotein FDH-O alpha subunit.

### Nucleotide sequence accession numbers. 

The sequences obtained by this whole-genome shotgun project have been deposited in DDBJ/EMBL/GenBank under the accession numbers CP011497, CP011498, CP011499, and CP011500.
